# [Corrigendum] Exogenous spermine attenuates diabetic kidney injury in rats by inhibiting AMPK/mTOR signaling pathway

**DOI:** 10.3892/ijmm.2026.6030

**Published:** 2026-10-29

**Authors:** Xinying Zhang, Li Zhang, Zhe Chen, Siwei Li, Bingbing Che, Ningning Wang, Junting Chen, Changqing Xu, Can Wei

Int J Mol Med 47: 27, 2021; DOI: 10.3892/ijmm.2021.4860

Following the publication of the above article and an Expression of Concern statement (doi: 10.3892/ijmm.2026.5954) after a reader drew the Editor's attention to the fact that the CD-2AP and podocin protein blots shown in the western blots in Fig. 3A on p. 6 were strikingly similar, suggesting that the same data had apparently been used to represent the two different proteins, the authors have now responded to our query. Upon investigating this figure, the authors have realized that the CD-2AP blot was inadvertently copied across to represent the podocin blot. However, since they had retained access to the original data, the authors are able to show this figure as was originally intended. The revised version of Fig. 3, now showing the correct data for the podocin blot, is shown on the next page. The authors confirm that the error made during the assembly of this figure did not have a significant impact on either the results or the conclusions reported in this study, and all the authors agree with the publication of this Corrigendum. The authors are grateful to the Editor of *International Journal of Molecular Medicine* for allowing them the opportunity to publish this Corrigendum; furthermore, they apologize to the readership of the Journal for any inconvenience caused.

## Figures and Tables

**Figure 3.**
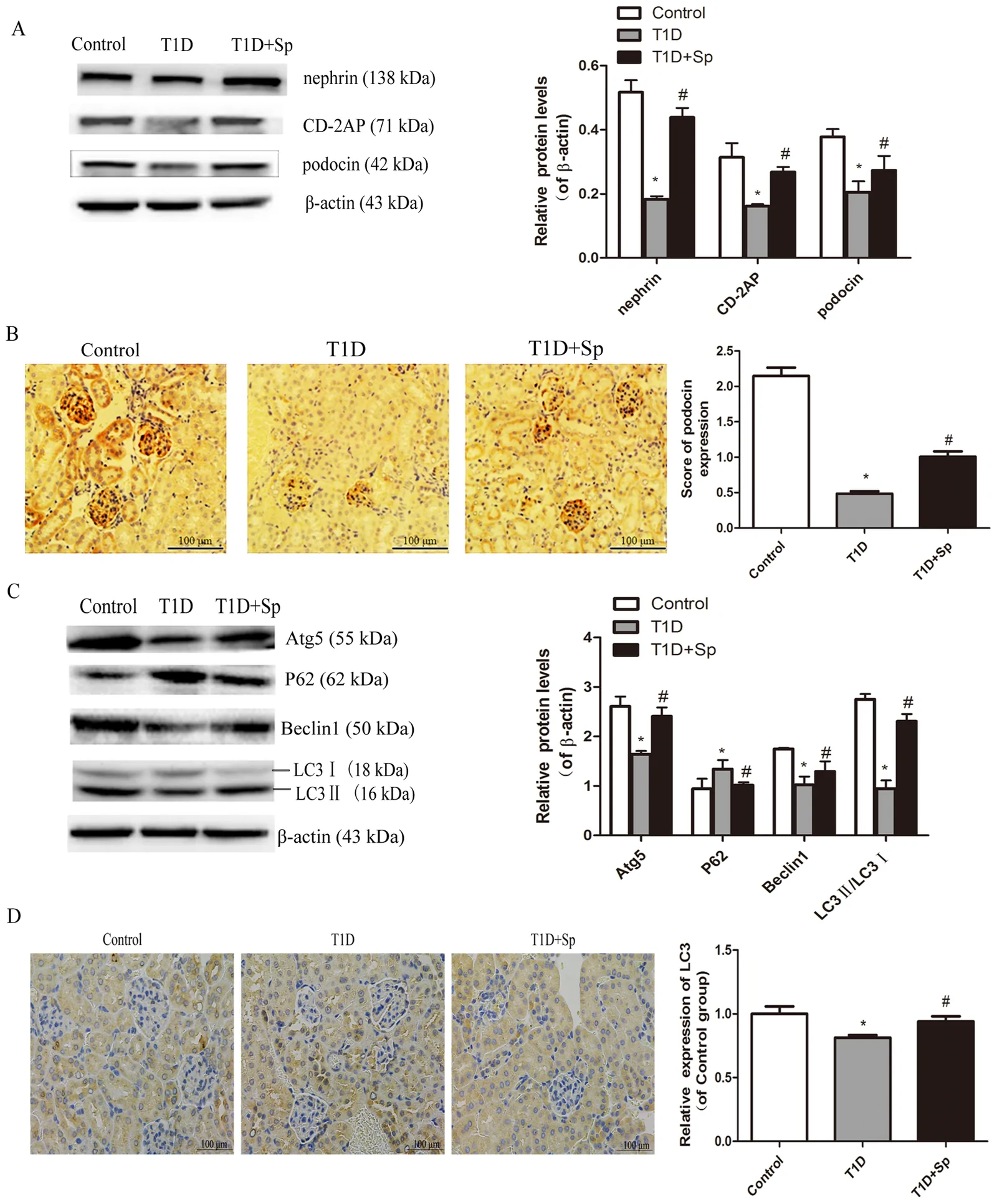
Spermine attenuates podocyte injury and promotes autophagy in T1D rats. (A) Representative immunoblotting of nephrin, CD2-AP and podocin protein levels in kidney sections. (B) Representative immunohistochemical staining for podocin in kidney sections (scale bar, 100 *μ*m) and its quantification. (C) Representative immunoblotting for Atg5, P62, Beclin1 and LC3II/LCI in kidney sections. (D) Representative immunohistochemical staining for LC3 in kidney sections (scale bar, 100 *μ*m) and its quantification. Data are expressed as the mean ± standard error of the mean (n=6). ^*^P<0.05 vs. the control group; ^#^P<0.05 vs. the T1D group. T1D, type 1 diabetic; CD-2AP, CD2-associated protein; Atg5, autophagy protein 5; LC3, microtube-associated proteins 1A/1B light chain 3; Sp, spermine-treated group.

